# A retrospective comparative study on the effects of denosumab combined with calcium and vitamin D versus calcium and vitamin D alone on bone remodeling markers in patients with osteoporotic fractures

**DOI:** 10.1097/MD.0000000000047540

**Published:** 2026-02-28

**Authors:** Zhijun Yang, Pengxi He, Qiang Xu

**Affiliations:** a Department of Orthopedics, Yan’an Hospital of Traditional Chinese Medicine, Yan’an City, Shaanxi Province, China; b Department of Traumatic Orthopedics, Affiliated Hospital of Yan’an University, Yan’an City, Shaanxi Province, China; c Department of Trauma Repair Surgery, Affiliated Hospital of Yan’an University, Yan’an City, Shaanxi Province, China.

**Keywords:** bone mineral density, bone remodeling, bone turnover markers, calcium, denosumab, osteoporotic fracture, P1NP, vitamin D, β-CTX

## Abstract

This study aims to compare and analyze the effects of denosumab combined with calcium and vitamin D versus calcium and vitamin D alone on bone remodeling markers and bone mineral density (BMD) in patients with osteoporotic fractures. This single-center retrospective comparative study included patients with osteoporotic fractures admitted between January 2022 and January 2024. According to the treatment regimen, patients were divided into the denosumab group (subcutaneous denosumab every 6 months plus oral calcium and vitamin D supplementation) and the calcium plus vitamin D (CaD) group (oral calcium and vitamin D supplementation only). Propensity score matching was used to balance baseline characteristics, with 60 cases in each group. Bone remodeling markers (β-isomerized C-terminal telopeptide of type I collagen [β-CTX], procollagen type I N-terminal propeptide [P1NP], bone-specific alkaline phosphatase, osteocalcin), BMD changes, and safety profiles were compared between the 2 groups before treatment, and at 6 and 12 months after treatment. After treatment, the levels of the bone resorption marker β-CTX and the bone formation marker P1NP were significantly reduced in the denosumab group, and remained significantly lower than those in the CaD group at all time points (time, group, and interaction effects all *P* < .001). After 12 months of treatment, BMD increases in the lumbar spine and femoral neck were 8.8% and 7.3% respectively in the denosumab group, significantly higher than in the CaD group (2.6% and 2.2%) (*P* < .01 for both group and interaction effects). Correlation analysis showed that in the denosumab group, the decreases in β-CTX and P1NP were significantly negatively correlated with BMD improvements in the lumbar spine and femoral neck (*r* = –0.33 to –0.42, *P* < .05). The overall incidence of adverse events did not differ significantly between the 2 groups (13.3% vs 8.3%, *P* > .05), and all events were mild. For patients with osteoporotic fractures, denosumab combined with calcium and vitamin D supplementation is more effective in suppressing bone turnover and improving bone mineral density than calcium and vitamin D alone, without increasing significant safety risks. Bone remodeling markers β-CTX and P1NP may serve as potential reference indicators for evaluating the therapeutic efficacy of denosumab.

## 1. Introduction

Osteoporosis is a systemic skeletal disorder characterized by decreased bone mass and microarchitectural deterioration of bone tissue, leading to increased bone fragility and susceptibility to fractures.^[1]^ Osteoporotic fractures, also known as fragility fractures, are the most severe complication of osteoporosis. Common sites include the vertebrae, hip, and distal radius. Among these, hip fractures are particularly concerning due to their high rates of disability and mortality, as well as their profound impact on patients’ quality of life, posing a substantial socioeconomic and healthcare burden on families and society.^[2,3]^

Bone remodeling is a fundamental physiological process that maintains skeletal homeostasis, involving osteoclast-mediated bone resorption and osteoblast-mediated bone formation. In postmenopausal osteoporosis, the sharp decline in estrogen levels disrupts the balance of bone remodeling, resulting in bone resorption significantly exceeding bone formation. This imbalance leads to progressive bone loss and deterioration of bone microarchitecture.^[4]^ Biochemical markers of bone turnover, such as β-isomerized C-terminal telopeptide of type I collagen (β-CTX), which reflects bone resorption, and procollagen type I N-terminal propeptide (P1NP), which reflects bone formation, can dynamically and sensitively assess bone metabolic status. These markers serve as valuable tools for monitoring the efficacy of anti-osteoporotic treatments.^[5]^

Calcium and vitamin D supplementation constitute the foundation of osteoporosis prevention and treatment. Adequate intake of calcium and vitamin D helps correct negative calcium balance, suppress secondary hyperparathyroidism, and provide substrates for bone matrix mineralization. However, calcium and vitamin D alone have limited effects in suppressing excessive bone resorption or markedly increasing bone mineral density (BMD).^[6]^ Therefore, for high-risk patients who have already sustained fractures, the combined use of potent anti-osteoporotic agents is essential.

Denosumab, a fully human monoclonal antibody, binds with high affinity to the receptor activator of nuclear factor kappa-B ligand (RANKL), thereby inhibiting the formation, activation, and survival of osteoclasts, resulting in potent suppression of bone resorption.^[7]^ Multiple large-scale randomized controlled trials (such as the FREEDOM study and its extension trials) have demonstrated that denosumab significantly reduces the risk of vertebral, nonvertebral, and hip fractures in postmenopausal women with osteoporosis and continuously increases lumbar spine and hip BMD for >10 years.^[8,9]^

However, in real-world clinical practice, evidence comparing the effects of denosumab plus calcium and vitamin D supplementation versus calcium and vitamin D alone on bone remodeling markers in patients with osteoporotic fractures remains limited. Real-world studies can complement randomized controlled trials by reflecting the actual therapeutic outcomes of different treatment strategies in routine clinical settings. Therefore, this retrospective comparative study aimed to investigate the dynamic changes in bone turnover markers (β-CTX, P1NP, bone-specific alkaline phosphatase [BALP], osteocalcin [OC]) and BMD in patients with osteoporotic fractures treated with denosumab combined with calcium and vitamin D versus calcium and vitamin D alone, as well as to evaluate the safety and correlations between these indicators, thereby providing clinical evidence for optimizing treatment strategies.

## 2. Materials and methods

### 2.1. General information

This study was approved by the Ethics Committee of Affiliated Hospital of Yan’an University (Approval No.: 2024-YAUH-kk194).This study was a single-center retrospective comparative study that collected clinical data of patients with osteoporotic fractures treated in our hospital from January 2022 to January 2024. All patients were diagnosed with osteoporotic fractures based on comprehensive clinical and imaging evaluations. According to treatment regimens determined by previous treatment timing, the patients were divided into 2 groups: the denosumab group and the calcium plus vitamin D group (CaD group). To minimize the influence of baseline differences on the study outcomes, propensity score matching (PSM) was performed using age, sex, fracture site, body mass index (BMI), and comorbid chronic diseases as matching variables. The matching ratio was set at 1:1 with a caliper value of 0.05. After PSM, 60 patients were included in each group. There were no statistically significant differences in baseline characteristics between the 2 groups (*P* > .05), indicating comparability.

### 2.2. Inclusion and exclusion criteria

*Inclusion criteria*: Patients aged ≥50 years, regardless of gender; met the diagnostic criteria outlined in the *Guidelines for the Diagnosis and Treatment of Primary Osteoporosis (2022 Edition*); had radiologically confirmed osteoporotic fractures (including vertebral, hip, distal radius, or other typical fragility fracture sites); received either denosumab treatment or CaD supplementation only and completed 12 consecutive months of standardized therapy; had complete clinical records and bone remodeling biochemical marker data (including β-CTX, P1NP, BALP, and OC), with clearly defined testing time points; had a follow-up duration of at least 6 months with good compliance.

*Exclusion criteria*: Secondary osteoporosis (e.g., hyperparathyroidism, Cushing syndrome, hyperthyroidism, diabetes mellitus, or bone metastases from malignant tumors); severe renal impairment (estimated glomerular filtration rate <30 mL/min·1.73 m^2^) or severe hepatic dysfunction; use of other anti-osteoporotic agents within the past 12 months (e.g., oral or intravenous bisphosphonates, teriparatide, romosozumab, raloxifene, etc); concurrent use of medications affecting bone metabolism during the study period (e.g., long-term or high-dose glucocorticoids, aromatase inhibitors, etc); incomplete clinical data or missing follow-up records.

### 2.3. Treatment

#### 2.3.1. Treatment regimens

*Denosumab group*: After fracture, patients in the denosumab group received subcutaneous injections of denosumab (trade name: Prolia®, 60 mg; administered subcutaneously in the abdominal region once every 6 months) during hospitalization or outpatient follow-up. In addition, they took calcium carbonate and vitamin D3 chewable tablets (II) (manufactured by Wyeth Pharmaceuticals Co., Ltd., containing 0.3 g of calcium and 60 IU of vitamin D3 per tablet), 2 tablets once daily.

*CaD group*: Patients in this group received only the same dosage of calcium carbonate and vitamin D3 chewable tablets (II) (2 tablets once daily) without any other anti-osteoporotic medication. Both groups were treated for a total of 12 months.

#### 2.3.2. Compliance assessment

To ensure the accuracy and comparability of the treatment interventions, patient medication compliance was systematically evaluated:

1. *Denosumab group*: Injection times were recorded using the hospital’s electronic medical records and drug management system to confirm that patients received denosumab injections at the prescribed intervals (once every 6 months). A delay exceeding 2 weeks was considered poor compliance.
2. *CaD group*: Patients or their family members recorded daily medication intake. During the follow-up period, researchers conducted telephone or outpatient follow-ups every 3 months to verify medication frequency and duration. Missing ≥20% of the cumulative prescribed doses was defined as poor compliance.

*Overall compliance criteria*: According to the World Health Organization formula for medication adherence:


$$\begin{array}{llll} & Compliance\;rate\left(\%\right)\; & =\left(Actual\;days\;of\;medication÷Planned\;days\;of\;medication\right)\; & \times 100\%. \end{array}$$

Patients with compliance ≥80% were classified as having good adherence.

### 2.4. Data collection

This study systematically collected the following clinical data and laboratory measurements:

#### 2.4.1. Demographic and clinical baseline data

Patient information, including age, sex, height, weight (used to calculate BMI), fracture site, comorbidities (such as hypertension and diabetes), and personal lifestyle history (including smoking and alcohol consumption), was obtained from the hospital’s electronic medical record system.

#### 2.4.2. Biochemical markers of bone metabolism

Fasting venous blood samples were collected in the early morning at 3 time points (before treatment [baseline], at 6 months, and at 12 months after treatment initiation). All samples were processed in the hospital’s central laboratory. Serum β-CTX and P1NP were measured using electrochemiluminescence immunoassay. OC levels were determined using enzyme-linked immunosorbent assay, while BALP activity was assessed via chemiluminescence assay.

To comprehensively evaluate calcium-phosphorus metabolic homeostasis, serum calcium (measured by the azo-arsen III method), phosphorus (phosphomolybdate method), alkaline phosphatase (continuous monitoring method), 25-hydroxyvitamin D [chemiluminescence method], and intact parathyroid hormone (electrochemiluminescence method) were also determined simultaneously.

#### 2.4.3. BMD measurement

BMD was measured before treatment and after 12 months of therapy using a dual-energy X-ray absorptiometer (Discovery Wi, Hologic Inc.). Measurements were performed by the same professionally trained technician to ensure consistency. Anteroposterior BMD values of the lumbar spine (L1–L4) and left femoral neck were recorded. The device was calibrated according to standard procedures before each measurement to ensure data accuracy and comparability.

#### 2.4.4. Safety and tolerability data

All adverse events occurring during the 12-month treatment period were thoroughly recorded through regular outpatient follow-up, telephone follow-up, and medical record review. These included hypocalcemia (defined as corrected serum calcium <2.10 mmol/L, hypocalcemia was defined as corrected serum calcium <2.10 mmol/L). Corrected calcium was calculated using the standard formula: corrected calcium (mmol/L) = measured calcium + 0.02 × (40 − serum albumin [g/L]), injection site reactions (e.g., erythema, induration), transient joint or muscle pain, and gastrointestinal discomfort. Each event’s occurrence, severity, management, and outcome were documented in detail.

### 2.5. Statistical analysis

All data analyses in this study were conducted using SPSS version 26.0 (IBM Corp., Armonk) statistical software. Measurement data conforming to a normal distribution were expressed as mean ± standard deviation ($\bar{x}$ ± s), and comparisons between groups were performed using the independent-samples *t* test. Categorical data were expressed as cases (percentages) [n (%)] and compared between groups using the χ^2^ test or Fisher exact test (when expected frequencies were <5).

To assess the temporal trends and intergroup differences in bone turnover markers (β-CTX, P1NP, BALP, and OC) and BMD before and after treatment, repeated measures analysis of variance (ANOVA) was applied. This analysis simultaneously evaluated the time effect (changes of indicators over time), group effect (differences between treatment groups), and interaction effect (whether the trends of change over time differed between groups).

To further explore the relationship between changes in bone metabolic markers and improvements in BMD during treatment, the change values (Δ) of each indicator were calculated as the difference between the baseline and 12-month measurements (Δ = 12-month value ‐ baseline value). Pearson correlation analysis was then used to examine the linear relationships between Δβ-CTX, ΔP1NP, ΔBALP, ΔOC, and ΔBMD (lumbar spine and femoral neck).

All statistical tests were two-tailed, and *P* < .05 was considered statistically significant.

## 3. Results

### 3.1. Comparison of baseline characteristics between the 2 groups

After propensity score matching, the baseline characteristics of the 60 patients in each group were balanced and comparable (see Table 1). Specifically, there were no significant differences between the 2 groups in mean age (denosumab group 69.1 years vs CaD group 67.4 years), gender distribution, BMI, or fracture site composition. At the same time, underlying diseases (such as hypertension and diabetes) and smoking or drinking history were also basically matched. Before treatment, the key indicators of bone remodeling markers, such as β-CTX (0.56 vs. 0.54 ng/mL) and P1NP (58.7 vs. 56.9 ng/mL), as well as bone mineral density of the lumbar spine and femoral neck, were at similar levels in both groups. These data indicate that the initial bone metabolic status of patients was consistent, providing a reliable basis for subsequent efficacy comparison.

**Table 1:** Baseline characteristics of patients in the 2 groups (±s, n = 60).

Variable	Denosumab group (n = 60)	CaD group (n = 60)	*t*/χ^2^ value	*P* value
Age (yr)	69.1 ± 8.3	67.4 ± 9.1	1.02	.31
Sex (male/female)	17/ 43	21/ 39	0.56	.456
BMI (kg/m^2^)	23.8 ± 3.6	22.9 ± 3.2	1.34	.183
Postmenopausal women [n (%)]	40 (93.0)	38 (90.5)	0.22	.641
Fracture site (n [%])				
Vertebral	26 (43.3)	21 (35.0)	0.9	.343
Hip	18 (30.0)	20 (33.3)	0.14	.707
Distal radius	9 (15.0)	11 (18.3)	0.23	.632
Others	7 (11.7)	8 (13.3)	0.08	.778
Comorbidities (n [%])				
Hypertension	23 (38.3)	25 (41.7)	0.14	.708
Diabetes mellitus	14 (23.3)	11 (18.3)	0.45	.504
Smoking history	10 (16.7)	8 (13.3)	0.24	.625
Alcohol consumption	6 (10.0)	9 (15.0)	0.58	.446
Laboratory parameters				
Serum calcium (mmol/L)	2.27 ± 0.12	2.25 ± 0.11	0.89	.377
Serum phosphorus (mmol/L)	1.13 ± 0.19	1.11 ± 0.18	0.59	.556
ALP (U/L)	83.4 ± 22.8	85.1 ± 24.6	0.37	.712
Osteocalcin (OC, ng/mL)	19.6 ± 7.2	20.1 ± 7.5	0.38	.705
25(OH)D (ng/mL)	21.8 ± 7.9	22.4 ± 8.2	0.4	.688
Parathyroid hormone (PTH, pg/mL)	45.6 ± 15.8	47.3 ± 16.1	0.54	.589
P1NP (ng/mL)	58.7 ± 19.6	56.9 ± 20.1	0.49	.626
β-CTX (ng/mL)	0.56 ± 0.20	0.54 ± 0.19	0.55	.583
Bone mineral density (BMD)				
Lumbar spine (L1–L4, g/cm^2^)	0.765 ± 0.087	0.758 ± 0.092	0.42	.676
Femoral neck (g/cm^2^)	0.645 ± 0.071	0.638 ± 0.075	0.5	.619

25(OH)D = 25-hydroxyvitamin D, ALP = alkaline phosphatase, CaD = calcium plus vitamin D, P1NP = procollagen type I N-terminal propeptide, β-CTX = β-isomerized C-terminal telopeptide of type I collagen.

### 3.2. Changes in bone remodeling markers

After treatment, the 2 groups showed different trends in bone remodeling markers (Table 2). The denosumab group exhibited a significant inhibitory effect on bone metabolism: β-CTX and P1NP levels were markedly reduced from baseline at 6 months and remained at low levels at 12 months (β-CTX: 0.21 ± 0.10–0.19 ± 0.09 ng/mL; P1NP: 34.2 ± 14.3–31.6 ± 13.1 ng/mL), both significantly lower than those of the CaD group at each time point. Repeated measures ANOVA showed that the time effect, group effect, and interaction effect for β-CTX and P1NP were all statistically significant (all *P* < .001). In contrast, there were no statistically significant differences in BALP and OC between the 2 groups at any time point.

**Table 2:** Comparison of bone turnover markers between the 2 groups before and after treatment and results of repeated-measures ANOVA.

Marker	Time point	Denosumab group (n = 60)	CaD group (n = 60)	Time effect *F (P*)	Group effect *F (P*)	Interaction effect *F (P*)
β-CTX (ng/mL)	Baseline	0.56 ± 0.20	0.54 ± 0.19	82.47 (<.001)	45.63 (<.001)	21.58 (<.001)
	6 months	0.21 ± 0.10	0.46 ± 0.17			
	12 months	0.19 ± 0.09	0.44 ± 0.18			
P1NP (ng/mL)	Baseline	58.7 ± 21.6	56.9 ± 20.1	69.85 (<.001)	32.14 (<.001)	18.92 (<.001)
	6 months	34.2 ± 14.3	52.7 ± 18.9			
	12 months	31.6 ± 13.1	50.9 ± 17.8			
BALP (U/L)	Baseline	18.7 ± 8.7	17.9 ± 6.1	1.38 (.253)	0.42 (.518)	0.66 (.517)
	6 months	17.2 ± 5.9	17.3 ± 6.0			
	12 months	16.8 ± 5.5	17.0 ± 5.7			
OC (ng/mL)	Baseline	19.1 ± 7.9	20.1 ± 7.5	2.21 (.115)	0.36 (.549)	0.91 (.403)
	6 months	17.3 ± 6.1	19.4 ± 6.8			
	12 months	16.8 ± 6.0	19.0 ± 6.5			

ANOVA = analysis of variance, BALP = bone-specific alkaline phosphatase, CaD = calcium plus vitamin D, OC = osteocalcin, P1NP = procollagen type I N-terminal propeptide, β-CTX = β-isomerized C-terminal telopeptide of type I collagen.

### 3.3. Improvement in BMD

After 12 months of treatment, bone mineral density improved in both groups; however, the increase was significantly greater in the denosumab group than in the CaD group (Table 3). In the denosumab group, lumbar spine BMD increased from 0.765 ± 0.087 g/cm^2^ to 0.832 ± 0.090 g/cm^2^ (an increase of 8.8%), and femoral neck BMD increased from 0.645 ± 0.071 g/cm^2^ to 0.692 ± 0.073 g/cm^2^ (an increase of 7.3%), both significantly higher than the 2.6% and 2.2% increases observed in the CaD group. Repeated measures ANOVA showed that the group effect and interaction effect for lumbar spine and femoral neck BMD were statistically significant (both *P* < .01).

**Table 3:** Comparison of bone mineral density (BMD) before and after treatment between the 2 groups.

Site	Time point	Denosumab group (n = 60)	CaD group (n = 60)	Time effect *F (P*)	Group effect *F (P*)	Interaction effect *F (P*)
Lumbar spine (L1–L4)	Baseline	0.765 ± 0.087	0.758 ± 0.092	**45.62 (<.001**)	**22.41 (<.001**)	**10.73 (.002**)
	12 months	0.832 ± 0.090	0.778 ± 0.089			
Femoral neck	Baseline	0.645 ± 0.071	0.638 ± 0.075	**38.17 (<.001**)	**18.92 (<.001**)	**9.56 (.003**)
	12 months	0.692 ± 0.073	0.652 ± 0.071			

CaD = calcium plus vitamin D.

### 3.4. Correlation between bone metabolism markers and BMD

**Figure 1.**
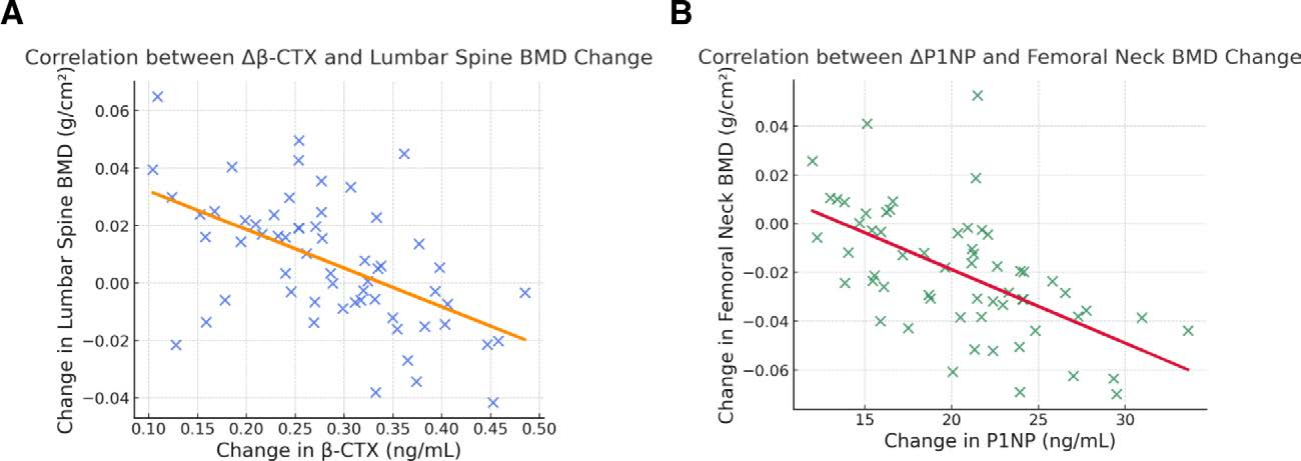
The correlation between bone metabolism indicators and bone density. (A) Correlation between the change in serum β-CTX and the change in lumbar-spine bone mineral density (BMD) after 12 months of treatment in the denosumab group. (B) Correlation between the change in serum P1NP and the change in femoral-neck BMD after 12 months of treatment in the denosumab group. P1NP = procollagen type I N-terminal propeptide, β-CTX = β-isomerized C-terminal telopeptide of type I collagen.

Analysis of posttreatment data showed that the decrease in the bone resorption marker β-CTX was negatively correlated with the increase in bone mineral density of the lumbar spine and femoral neck (Table 4 and Fig. 1A). Similarly, the reduction in the bone formation marker P1NP was also negatively correlated with the improvement in BMD at these 2 sites (Table 4 and Fig. 1B). In contrast, changes in BALP and OC showed no significant correlation with changes in bone mineral density.

**Table 4:** Correlation between changes in bone turnover markers and changes in BMD.

Variable	Lumbar spine BMD change (*r*, *P*)	Femoral neck BMD change (*r*, *P*)
Δβ-CTX	**–0.42 (*P* = .003**)	**–0.39 (*P* = .005**)
ΔP1NP	**–0.36 (*P* = .008**)	**–0.33 (*P* = .012**)
ΔBALP	–0.15 (*P* = .258)	–0.12 (*P* = .317)
ΔOC	–0.18 (*P* = .197)	–0.16 (*P* = .221)

BALP = bone-specific alkaline phosphatase, OC = osteocalcin, P1NP = procollagen type I N-terminal propeptide, β-CTX = β-isomerized C-terminal telopeptide of type I collagen.

### 3.5. Treatment safety

During the treatment period, a total of 8 patients (13.3%) in the denosumab group reported adverse events, including 3 cases of mild hypocalcemia, 2 cases of injection-site erythema, 2 cases of transient joint or muscle pain, and 1 case of gastrointestinal discomfort (Table 5). In the CaD group, 5 patients (8.3%) experienced adverse events, including 4 cases of gastrointestinal discomfort and 1 case of joint or muscle pain. There was no statistically significant difference in the overall incidence of adverse reactions between the 2 groups, and all adverse events were mild and resolved after symptomatic management.

**Table 5:** Adverse events during treatment in the 2 groups (n, %).

Adverse event	Denosumab group (n = 60)	CaD group (n = 60)	*χ*^2^ value	*P* value
Mild hypocalcemia	3 (5.0)	0 (0)	1.84	.175
Injection-site erythema	2 (3.3)	–	–	–
Transient joint or muscle pain	2 (3.3)	1 (1.7)	0.34	.559
Gastrointestinal discomfort	1 (1.7)	4 (6.7)	1.78	.182
Total adverse events	8 (13.3)	5 (8.3)	0.72	.396

CaD = calcium plus vitamin D.

## 4. Discussion

Through a retrospective comparative analysis with baseline data balanced by PSM, this study evaluated the effects of denosumab combined with calcium and vitamin D versus calcium and vitamin D alone on bone metabolism and BMD in patients with osteoporotic fractures. Our main findings demonstrated that, compared with basic supplementation therapy, denosumab in combination with calcium and vitamin D more rapidly and effectively suppressed bone turnover, resulting in significant improvements in BMD. Moreover, the degree of BMD improvement was closely associated with the extent of suppression of bone remodeling markers, while the treatment exhibited good safety and tolerability.

### 4.1. Effects on bone remodeling markers

This study observed that denosumab significantly and rapidly reduced the levels of the bone resorption marker β-CTX and the bone formation marker P1NP within 6 months, and this effect was maintained at 12 months. This finding is fully consistent with the pharmacological mechanism of denosumab as a potent RANKL inhibitor. By blocking RANKL, a key signal required for osteoclast activation and survival, denosumab directly suppresses excessive bone resorption. The subsequent reduction in bone formation (reflected by the decrease in P1NP) represents the “coupling” mechanism of bone remodeling (when bone resorption is strongly inhibited, bone formation activity correspondingly decreases).^[10]^ This aligns with results from pivotal randomized controlled trials, confirming that denosumab can effectively reverse the high bone turnover state even in real-world fracture patient populations.

In contrast, patients in the CaD group showed only slight decreases in β-CTX and P1NP, suggesting that while calcium and vitamin D supplementation can provide mineral substrates and mildly reduce secondary hyperparathyroidism, it is insufficient to effectively counteract the pathological high bone turnover characteristic of osteoporosis.^[11]^

It is noteworthy that in this study, BALP and OC showed no significant differences between the 2 groups. This may be attributed to the biological properties of these markers. BALP reflects early osteoblastic activity and may be less sensitive to short-term changes than P1NP.^[12]^ Although OC is synthesized by osteoblasts, most of it becomes incorporated into the bone matrix. Its serum levels are influenced by both bone formation rate and mineralization rate, and its degradation and clearance mechanisms are complex, which may explain its less direct and less pronounced response to treatment compared with P1NP.^[13]^

### 4.2. Effects on BMD

After 12 months of treatment, the increases in lumbar spine and femoral neck BMD in the denosumab group were significantly greater than those in the CaD group. This result provides strong evidence that by potently suppressing bone turnover, denosumab can markedly increase bone mass. The mechanism lies in the fact that when denosumab substantially reduces the rate of bone resorption, bone remodeling units that would otherwise be excessively resorbed are preserved. As the depth of resorption cavities decreases in each remodeling cycle while bone formation remains relatively unchanged, a positive balance is achieved, leading to a net gain in bone mass.^[14]^ The greater increase in lumbar spine BMD (8.8%) compared with that of the femoral neck (7.3%) may be attributed to the higher bone turnover rate in axial bones (mainly trabecular bone) than in peripheral bones (with a higher cortical bone proportion), resulting in a faster and more pronounced response to potent antiresorptive therapy.^[15]^ The modest increases observed in the CaD group (2.6% and 2.2%) may be related to natural recovery of bone mass after immobilization due to fracture and to calcium and vitamin D ensuring normal bone mineralization, but their magnitude is far below the therapeutic benefit achieved with pharmacologic intervention.^[16]^

### 4.3. Correlation between bone metabolic markers and BMD

An important finding of this study was that in the denosumab group, the reductions (Δ values) in the bone resorption marker β-CTX and the bone formation marker P1NP were significantly negatively correlated with the increases in lumbar spine and femoral neck BMD. This finding has important clinical implications: the greater the suppression of bone turnover during denosumab treatment, the higher the subsequent gain in bone mass. This observation aligns with quantitative theoretical models of bone remodeling, which posit that reducing bone turnover and remodeling space itself increases bone mass, while stronger inhibition results in less bone loss and a more pronounced positive bone balance.^[11]^ These results support the potential use of β-CTX and P1NP as early (e.g., 3–6 months) response biomarkers during denosumab therapy to predict long-term BMD outcomes, helping clinicians to identify poor responders early and adjust treatment strategies accordingly.^[17,18]^ The lack of significant correlation between BALP and OC changes and BMD improvement further suggests that β-CTX and P1NP are more sensitive and reliable indicators for monitoring the efficacy of denosumab therapy.

### 4.4. Treatment safety and tolerability

During the 12-month treatment period, there was no statistically significant difference in the overall incidence of adverse events between the 2 groups, and most events were mild and transient. The 3 cases of mild hypocalcemia reported in the denosumab group highlight the importance of maintaining adequate calcium and vitamin D intake before and during potent antiresorptive therapy, particularly with denosumab (an aspect emphasized in all relevant clinical guidelines). No serious adverse events occurred in either group, confirming that denosumab combined with calcium and vitamin D is safe and well tolerated in patients with osteoporotic fractures under real-world clinical conditions.

### 4.5. Study limitations

This study has several limitations. First, this study was retrospective in nature, and the sample size was determined by the number of eligible patients treated during the study period. Therefore, an a priori sample size calculation was not feasible, which is an inherent limitation of retrospective real-world studies. Although PSM was employed to reduce confounding bias, the inherent limitations of retrospective design (such as the influence of unmeasured confounders) cannot be completely ruled out. Second, the sample size was relatively small, and the follow-up period was limited to 12 months, preventing evaluation of long-term efficacy, safety, and fracture risk reduction. Future multicenter, large-sample, and longer-term prospective studies are needed to further validate our findings.

## 5. Conclusion

The results of this retrospective analysis indicate that in patients with osteoporotic fractures, denosumab combined with calcium and vitamin D demonstrates stronger suppression of bone turnover and a more pronounced improvement in bone mineral density within 12 months compared with calcium and vitamin D supplementation alone. Additionally, the observed correlations between changes in bone resorption and formation markers (β-CTX and P1NP) and improvements in bone mineral density suggest their potential value as indicators for monitoring treatment efficacy. Both treatment regimens showed acceptable tolerability during the study period. These findings provide real-world clinical evidence supporting the use of denosumab in patients with osteoporotic fractures; however, its long-term efficacy and safety require further verification through larger, prospective studies.

## Author contributions

**Conceptualization:** Zhijun Yang, Pengxi He, Qiang Xu.

**Data curation:** Zhijun Yang, Pengxi He, Qiang Xu.

**Formal analysis:** Zhijun Yang, Pengxi He, Qiang Xu.

**Funding acquisition:** Pengxi He, Qiang Xu.

**Investigation:** Pengxi He, Qiang Xu.

**Writing – original draft:** Zhijun Yang, Pengxi He, Qiang Xu.

**Writing – review & editing:** Zhijun Yang, Pengxi He, Qiang Xu.
